# Current outcomes of triceps-sparing elbow arthroplasties: a systematic review

**DOI:** 10.1016/j.xrrt.2025.100604

**Published:** 2025-10-28

**Authors:** James R. Satalich, Shahabeddin Yazdanpanah, Grayson M. Talaski, Devi-Preetham Veeramgari, Nicholas R. Kiritsis, John W. Cyrus, Benjamin P. Cassidy, Matthew S. Smith, Jennifer L. Vanderbeck

**Affiliations:** a Department of Orthopaedic Surgery, Virginia Commonwealth University, Richmond, VA, USA; b College of Medicine, Northeast Ohio Medical University, Rootstown, OH, USA; c Department of Orthopedics and Rehabilitation, University of Iowa, Iowa City, IA, USA

**Keywords:** Triceps, Triceps sparing, Elbow arthroplasty, Total elbow arthroplasty, Elbow hemiarthroplasty, Range of motion, Complications

## Abstract

**Background:**

Total elbow arthroplasty (TEA) is a reconstructive option for fractures, arthritis, and tumors, particularly in older adults. Approaches for the procedure can be categorized into triceps-sparing (TS) or triceps-detaching (TD). TD approaches are well-studied, but are associated with many complications such as infections, limited range of motion, and triceps insufficiency. Despite being more technically demanding, TS approaches are rising in popularity due to preservation of the triceps insertion. This study consolidates the most recent literature on TS-TEAs and TS elbow hemiarthroplasties (EHAs) to better inform updated clinical decision-making.

**Methods:**

A PROSPERO-registered systematic review was conducted on March 10, 2025, searching PubMed, Ovid-Embase, MEDLINE, and Web of Science for studies published since 2020 that investigate TS elbow arthroplasty findings in adults. Extracted outcomes included demographics, range of motion metrics, and complications. Frequency-weighted means, t-tests, and chi-square tests were accordingly utilized in R Studio for statistical analyses.

**Results:**

Eight moderate-quality retrospective studies were identified (5 TS-TEA and 3 TS-EHA) out of 290. Patients (n = 252; TS-TEA 195; TS-EHA 57) had average ages of 64.5 ± 12.5 and 73.1 ± 10.6, with average follow-ups of 43.8 ± 39.5 and 56.3 ± 20.0 months for TS-TEA and TS-EHA, respectively. Average flexion degrees were 129.7° ± 19.5 and 129.0° ± 13.8, followed by extension deficits at 17.9° ± 17.3 and 19.8° ± 12.9, respectively. Average postoperative prosupination arc ranges were 140.6°-144.6° and 155°-171°, respectively. Complication rates were 22.9% after TS-TEA and 54.4% after TS-EHA, characterized by literature-compared high rates of ulnar nerve symptoms and heterotopic ossification, but low rates of infection and triceps insufficiency.

**Conclusion:**

TS approaches, either in TEA or EHA, are reasonable options for surgeons today. Further studies are recommended for more granular and consistent elucidation, with arthroplasty approaches remaining individualized to patient needs and surgeon preferences.

Elbow arthroplasty is frequently indicated for conditions such as rheumatoid arthritis, osteoarthritis, fractures, and tumors.^4^^,^^9^^,^^17^^,^^20^, ^21^, ^22^^,^^45^ Approaches for both total elbow arthroplasty (TEA) and elbow hemiarthroplasty (EHA) are often categorized as either triceps-detaching (TD) or triceps-sparing (TS).^4^ Historically, TD approaches like the Bryan-Morrey triceps reflecting technique have offered excellent joint exposure, making them an effective strategy in treating elbow pathology.^4^^,^^6^^,^^13^ However, the use of TD approaches has been linked to high complication rates approaching 45%, failures and revision rates up to 35%, and instances of triceps insufficiency that may contribute to range-of-motion (ROM) hindrances.^8^^,^^12^^,^^24^^,^^26^^,^^37^

TS approaches, also called “triceps-preserving”, “triceps-retaining”, or “triceps-on”, first discussed by Dr. Bernard F. Morrey and associates, were created to preserve the triceps insertion, minimize triceps failure, and theoretically improve joint ROM.^4^^,^^5^^,^^8^^,^^27^ However, TS approaches are perceived as more technically challenging due to limited comparative exposure and difficulties with component alignment.^4^^,^^8^^,^^16^^,^^19^ Nonetheless, with higher complication rates and triceps failures in TD approaches, TS approaches have gained popularity among elbow specialists.^4^^,^^8^^,^^9^^,^^24^^,^^27^

To date, no systematic review has synthesized the most current literature on TS elbow arthroplasties and their outcomes. Therefore, the purpose of this study is to consolidate and evaluate the postoperative outcomes following TS-TEAs and TS-EHAs published since 2020, including patient profiles, ROM metrics, and complication-related data. By assessing these findings, the present review aims to provide broadened awareness regarding TS-approach viability for physicians and patients considering elbow replacement, bolstering clinical shared decision-making and inviting avenues for future research.

## Methods

### Study creation and initial search

This systematic review was conducted in accordance with the Preferred Reporting Items for Systematic Reviews and Meta-Analyses guidelines.^29^ The study protocol was registered in PROSPERO prior to article screening (CRD420251004022). The initial literature search was carried out across 5 databases—PubMed, MEDLINE, Ovid-Embase, Web of Science, and Cochrane—encompassing all records retrieved by the search algorithm from January 2020 up to March 10, 2025. The search algorithm used in this study was, “(elbow or elbows) AND (replacement OR replacements OR arthroplasty OR arthroplasties OR reconstruction OR reconstructions OR implant OR implants OR prosthesis OR prostheses) AND (triceps OR "triceps sparing" OR "tricep sparing" OR "tricep on" OR "triceps on" OR morrey OR morrey's)”.

### Eligibility criteria

Inclusion criteria were articles that examined outcomes following triceps-sparing TEA or EHA in adult patients, including observational studies, randomized controlled trials, and articles with full text. Exclusion criteria were nonhuman or cadaveric articles, case series with less than 10 patients, non-English articles, technical notes, letters to editor, surveys, previous systematic reviews, meta-analyses, and abstracts.

### Article screening process

Articles were located using the previously mentioned search criteria in 5 databases, all retrieved articles were imported into Rayyan.ai: a widely used online software for efficient article screening.^28^ Duplicate articles were initially removed manually, and the remaining articles were then screened by title and abstract according to the inclusion and exclusion criteria. The screening process was conducted by 2 authors. After the title and abstract screening, the full texts of the remaining articles were reviewed to determine final inclusion.

### Data extraction

Data extraction was performed by 2 independent authors. Data extracted from the included articles included first author, year of publication, study type, number of patients, demographic information, preoperative and postoperative ROM metrics, patient-reported outcome metrics, functional outcomes, follow-up duration, complications, and additional outcomes.

### Article quality grading

All observational studies were evaluated using the Methodological Index for Nonrandomized Studies (MINORS), a tool frequently applied in systematic reviews and meta-analyses.^33^ The MINORS scale distinguishes between comparative and noncomparative studies, scoring comparative studies out of 24 points and noncomparative studies out of 16 points. Each item on the MINORS scale is rated from 0 to 2 points, reflecting the quality of the study. Articles were categorized as “high-quality”, “moderate-quality”, or “low-quality” based on their scoring and comparative/noncomparative nature. For comparative studies, high-quality articles scored 24 points, moderate-quality articles scored 15-23 points, and low-quality articles scored less than 15 points. For noncomparative studies, high-quality articles scored 16 points, moderate-quality articles scored 10-15 points, and low-quality articles scored less than 10 points.^18^ All grading was completed by 2 authors.

### Statistical analysis

Statistical analysis was performed using RStudio software version 2023.06.1 + 524 (R Foundation for Statistical Computing, Vienna, Austria). Meta-analyses and more advanced statistics were deemed inappropriate in the present setting due to reporting heterogeneity and a lack of appropriate inter-study comparisons and data. Therefore, an alternative narrative approach was pursued, comprised of descriptive statistics and frequency-weighted means (FWMs) to quantify syntheses. If setting-appropriate, 2-tailed t-tests were run to evaluate any significant differences between preoperative and postoperative patient outcomes. Statistical significance was denoted as *P* value <.05.

## Results

### Initial search results

**Figure 1:**
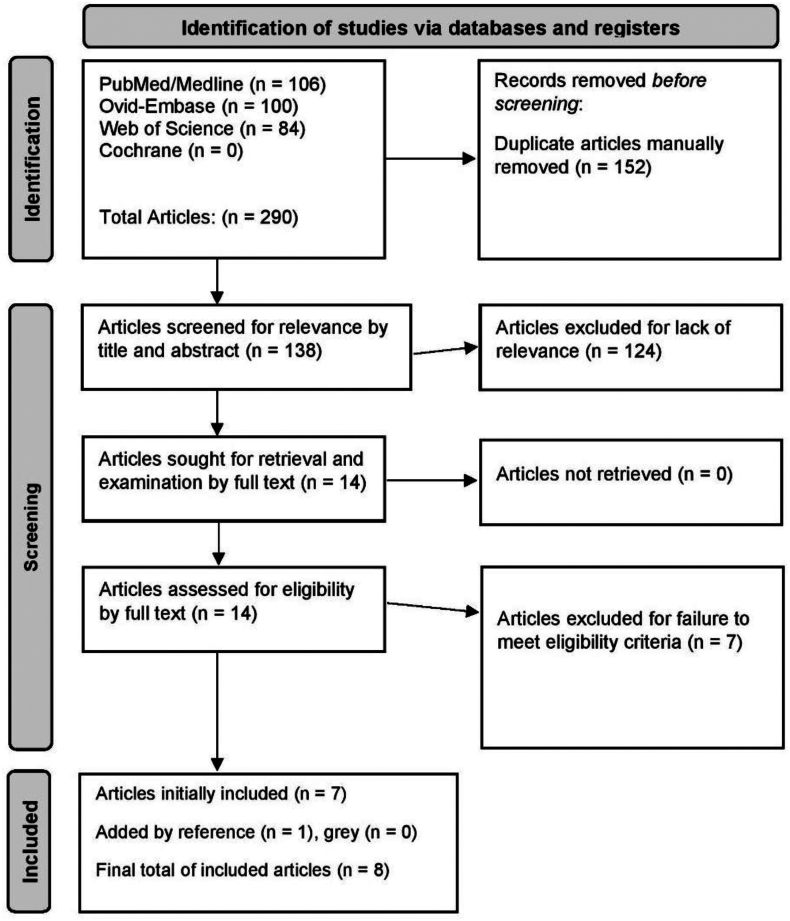
The Preferred Reporting Items for Systematic Reviews and Meta-Analyses (PRISMA) diagram, outlining the search process and final inclusions.

The database search resulted in 290 articles: 152 after removing duplicates. Fourteen articles remained after title and abstract screening for full text examination, of which 7 met the eligibility criteria. The reference search yielded 1 additional study, with zero coming from the gray literature search. Finally, a total of 8 articles met the inclusion criteria and were selected for data extraction (Fig. 1).^1^^,^^14^^,^^15^^,^^23^^,^^32^^,^^36^^,^^38^^,^^44^

### Article quality results

All 8 included articles were observational and retrospective in nature. The average MINORS score for the present study was 11.1 ± 0.3. Each study in this systematic review received a "moderate-quality" MINORS classification (Table I).^33^

**Table I:** The Methodological Index for Nonrandomized Studies grading for all included articles.

Author (Yr)	Total MINORS score	Clearly stated aim	Inclusion of consecutive patients	Prospective collection of data	End points appropriate to study aim	Unbiased assessment of study end point	Follow-up period appropriate to study aim	Less than 5% lost to follow up	Prospective calculation of the study size	Adequate control group	Contemporary groups	Baseline equivalence of groups	Adequate statistical analysis
Meijering (2022)^23^	11	2	2	0	2	2	2	1	0	0	0	0	0
Kahan (2022)^15^	12	2	2	0	2	2	2	2	0	0	0	0	0
Zhang (2023)^44^	11	2	2	0	2	1	2	2	0	0	0	0	0
Ahmed (2024)^1^	11	2	2	0	2	1	2	2	0	0	0	0	0
Prada (2025)^32^	11	2	2	1	2	2	1	1	0	0	0	0	0
Jenkins (2021)^14^	11	2	2	0	2	1	2	2	0	0	0	0	0
Taylor (2021)^36^	11	2	2	0	2	1	2	2	0	0	0	0	0
Virani (2025)^38^	11	2	2	0	2	1	2	2	0	0	0	0	0

*MINORS*, Methodological Index for Nonrandomized Studies.

### Patient and study characteristics

In the TS-TEA group (n = 195), the FWM age was 64.5 ± 12.5 years, with a follow-up of 43.76 ± 39.5 months (80% study reporting). For the TS-EHA group (n = 57), the FWM age was 73.1 ± 10.6 years, and follow-up of 56.3 ± 20.0 months. Notably, the TS-EHA group was significantly older than the TS-TEA group (*P* = .002). Most patients were female: 68.7% in TS-TEA and 73.7% in TS-EHA. Individual study demographics are detailed in addition to these composite values in Table II.

**Table II:** Demographics table for included studies.

Author (Yr)	TS elbow arthroplasty procedure: TEA or EHA	Patients (N)	Average age (SD or range)	Average follow-up (SD or range) in months	Female %
Meijering (2022)^23^	TEA	46	64.7 (13.6)	/	56.5
Kahan (2022)^15^	TEA	53	61.8 (13.1)	30.2 (22.7)	81
Zhang (2023)^44^	TEA	23	66.1 (15.3)	92.6 (52-136)	69.6
Ahmed (2024)^1^	TEA	38	67	66 (36-108)	57.9
Prada (2025)^32^	TEA	35	64 (10.2)	7.9 (4.8)	77.1
Jenkins (2021)^14^	EHA	37	75 (29-93)	61 (24-105)	70.3
Taylor (2021)^36^	EHA	8	72.1 (9.1)	29.9 (11.4-58.8)	100
Virani (2025)^38^	EHA	12	67.75 (10.2)	59.4 (19.7)	66.7

*EHA*, elbow hemiarthroplasty; *TEA*, total elbow arthroplasty; *SD*, standard deviation.

A slash (/) is an indication that the data were not reported.

### Range of motion outcome measures

Postoperatively, the FWM elbow flexion in the TS-TEA group was 129.7° ± 19.5 (60% reporting), with an extension deficit of 17.9° ± 17.3 (60% reporting). In the TS-EHA group, flexion averaged 129° ± 13.8, and extension deficit was 19.8° ± 12.9. Pronation and supination in the TS-EHA group averaged 78.1° ± 9.2 and 80.4° ± 8.9, respectively. For the TS-TEA group, only Prada et al (2025) reported separate pronation and supination values: 73.3° ± 19.3 and 71.3° ± 25.4, respectively.^32^ Additionally, pronation and supination values were summated to report elbow rotation as prosupination arc for comparison, following the method described by Morrey et al (1981).^25^ The TS-TEA group showed an average prosupination arc ranging from 140.6°-144.6° (40% reporting), while the TS-EHA group ranged from 155°-171°. Individual study outcomes are provided in addition to these composite values in Table III. For consistency in presentation and reporting, ROM data from Zhang et al (2023) were combined using the group-specific means and standard deviations; however, flexion arc was not extracted into separated flexion and extension degrees due to a lack of such reporting in the study (Table III).^44^ Furthermore, it should be noted that for calculations, study-reported extension degree and extension deficit were used interchangeably due to synonymity for greater homogeneity of data reporting.^23^^,^^38^

**Table III:** Postoperative ROM outcomes for included studies.

Author (Yr)	TS elbow arthroplasty procedure: TEA or EHA	Average elbow flexion or flexion arc degree (SD or range)	Average elbow extension degree or deficit (SD or range)	Average pronation degree (SD or range)	Average supination degree (SD or range)	Average prosupination arc (SD), or CPS arc
Meijering (2022)^23^	TEA	127.4 (12.7)	27.3 (19.5)	/	/	/
Kahan (2022)^15^	TEA	136.8 (9.6)	8.3 (10.7)	/	/	/
Zhang (2023)^44^	TEA	Flexion arc 98.9 (25.9)	/	/	/	140.6 (16.3)
Ahmed (2024)^1^	TEA	/	/	/	/	/
Prada (2025)^32^	TEA	121.8 (31.4)	20.2 (14.6)	73.3 (19.3)	71.3 (25.4)	144.6
Jenkins (2021)^14^	EHA	132 (100-150)	19 (0-50)	74 (30-90)	81 (50-90)	155
Taylor (2021)^36^	EHA	135 (9)	21 (15)	87 (5)	84 (9)	171
Virani (2025)^38^	EHA	115.8 (8.2)	21.7 (11.1)	85 (4.9)	76.3 (7.3)	161.2

*EHA*, elbow hemiarthroplasty; *TEA*, total elbow arthroplasty; *SD*, standard deviation; *ROM*, range of motion.

“CPS Arc” refers to “combined pronation + supination Arc”, as previously described. A slash (/) is an indication that the data were not reported.

### Complications

Complication rates ranged from 7.9% to 43.8% for TS-TEA studies, with a FWM of 22.9%. For TS-EHA studies, rates ranged from 18.9% to 83.3%, averaging 54.4%. The most frequently reported complications were ulnar nerve symptoms (TS-TEA 10.5% average, range 2.9%-17.4%, 60% reporting; TS-EHA 18.9% average, range 12.5%-21%) and heterotopic ossification (TS-TEA 5.2% average, range 2.9%-8.7%, 40% reporting; TS-EHA 32.2% average, range 21%-66.7%, 66.7% reporting). Infections rates for TS-TEAs were reported at a FWM of 2.9%, ranging from 1.9% to 4.3%, with 60% reporting; no infections were reported for TS-EHAs. Triceps extensor failure was only reported in one TS-TEA study at a FWM of 1.9%, with none in the TS-EHA cohort. Less consistently reported complications, as well as individual study-level data, are presented in Table IV. Notably, in some cases complications were not reported per patient, which may result in cumulative percentages exceeding 100%.

**Table IV:** Detailing of complications and complication rates for included studies.

Author (year)	TS elbow arthroplasty procedure: TEA or EHA	Total complications (%)	Ulnar nerve symptoms (%)	Heterotopic ossification (%)	Other complications (%)
Meijering (2022)^23^	TEA	43.8	17.4	/	Radial nerve neuropathy (2.2), olecranon bursa osseous fragment (2.2), posterior interosseous nerve neuropathy (2.2), aseptic loosening (2.2), infection (4.3), dislocation (13.0)
Kahan (2022)^15^	TEA	20.8	/	/	Wound issues (9.4), triceps extensor failure (1.9), revision (1.9), aseptic loosening (1.9), infection (1.9), fracture (3.8)
Zhang (2023)^44^	TEA	17.4	8.7	8.7	/
Ahmed (2024)^1^	TEA	7.9	/	/	Revision (2.6), aseptic loosening (5.3), infection (2.6)
Prada (2025)^32^	TEA	18.9	2.9	2.9	Revision (8.6), fracture (2.9)
Jenkins (2021)^14^	EHA	51.3	21	21	Wound issues (2.7), revision (2.7), dislocation (5.4)
Taylor (2021)^36^	EHA	25	12.5	/	Dislocation (12.5)
Virani (2025)^38^	EHA	83.3	16.7	66.7	Nonunion (41.7)

*TS*, triceps-sparing; *EHA*, elbow hemiarthroplasty; *TEA*, total elbow arthroplasty.

## Discussion

The objective of this systematic review was to detail the postoperative outcomes of TS elbow arthroplasty, both total and hemi procedures, in order to evaluate the approach's viability. The present study found a FWM extension deficit of 17.9° after TS-TEA and 19.8° after TS-EHA. In the absence of a dedicated systematic review of TD approaches, this is compared to Stoddart et al (2024), which pooled data predominantly from TD approaches, reporting mean extension deficits of approximately 24.4° after TEA and 21.4° after EHA.^35^ The modest improvement in reduced extension deficit can likely be attributed to both the muscular anatomy involved and the extensor mechanism preservation of a TS approach, thus validating the presumption of the approach improving patient elbow ROM.^4^^,^^40^

Regarding elbow flexion, our results determined a FWM of 129.7° and 129° after TS-TEA and TS-EHA, respectively. For comparison, TD-approach studies have reported mean elbow flexions of 130° and 123.4° after TD-TEA and TD-EHA, respectively.^11^^,^^35^ The similarity between TS and TD approaches in postoperative elbow flexion likely reflects the fact that neither technique alters the elbow flexors such as the brachioradialis and biceps, and thus minimal contribution from the triceps results in the observed flexion values all within the intervals of acceptability in the literature.^25^^,^^40^^,^^41^

In terms of forearm rotation, this outcome has been determined in the literature by combining pronation and supination values to yield a prosupination arc: a method adopted in both the present study and in the included articles when reporting individual ROM data.^25^ The present study's prosupination arc findings ranged from 140.6° to 144.6° (40% reporting) after TS-TEA and 155° to 171° after TS-EHA. In TD-approach literature, prosupination arcs have been reported from 122° to 160° and 137° to 179° after TD-TEA and TD-EHA, respectively.^8^^,^^11^^,^^31^^,^^43^ This broad range may reflect sample size limitations and the presence of outliers; however, intertechnique comparative postoperative and pathological stiffness cannot be excluded as contributing factors, which have been noted in prior elbow arthroplasty literature.^7^ Even so, the present findings pertaining to TS-approach outcomes for prosupination arc fall within the literature-reported established ranges for TD-approach elbow arthroplasty.

The FWM complication rate in the TS-TEA cohort was 22.9%. When compared to TD-approach literature with complication rates ranging from 24.3% to 36.1%, it appears that TS-TEAs have a modestly lower comparative complication rate.^27^^,^^35^^,^^39^ This difference may be attributed to the amount of exposure, reduction in soft tissue disruption, and preservation of the triceps insertion.^4^ When examining individual complications, the present study's heterotopic ossification rate of 5.2% aligns closely with that of TD-TEA literature at 3.6%.^35^ Interestingly though, ulnar nerve complications were lower in TD-TEA literature than in the present study, with reported rates of around 3% compared to TS-TEA in the present study at 10.5%.^8^^,^^39^^,^^41^ This may reflect the evolutionary history of surgical approaches, as TD-TEAs did not always have low ulnar nerve complication rates, which highlights the need for further optimization of the TS technique as it gains popularity.^39^ Notably, the FWM infection rate of 2.9% and triceps insufficiency rate of 1.9%, though infrequently reported, were marginally lower than the 8% infection rate and 4.9% triceps insufficiency rate in TD-TEA literature.^24^^,^^35^ The observed differences may be presumed to be due to the aforementioned advantage of the TS approach in preserving the triceps insertion and vascular anatomic layers between the implant and the skin incision, thereby reducing potential infection and muscular insufficiency risk.^4^^,^^34^

For TS-EHAs, the FWM complication rate was 54.4%. Compared to a literature-defined TD-EHA mean complication rate of 28.1%, the present study's findings are remarkably higher.^35^ However, this finding should be interpreted with caution, as distinctions between major and minor complications remain inconsistently defined in elbow arthroplasty literature, with similar consideration for instances where multiple complications are reported within the same patient.^31^^,^^42^ Notably, heterotopic ossification emerged as a prominent complication in the present study's TS-EHA cohort, with a FWM of 32.2% (66.7% reporting). In comparison, this finding is debated within in the literature, as TD-EHA studies have reported ranges from 0% to 40% across both reviews and individual studies.^2^^,^^8^^,^^11^^,^^30^^,^^31^^,^^35^ It has been suggested that such elevated rates may reflect the natural incidence of heterotopic ossification in response to specific pathological conditions, implicating an underlying process rather than the surgical technique or operation itself.^10^^,^^31^ Furthermore, differences in how elbow arthroplasty studies categorize heterotopic ossification and stiffness as separate complications may also influence the reported rates and add complexity to comparisons made.^8^^,^^35^

When interpreting the findings of the present systematic review, several limitations must be considered. First, this study is constrained by a relative lack of recent literature describing TS elbow arthroplasties, as evidenced by the identification of only 5 TEA and three EHA studies meeting inclusion criteria. Additionally, elbow arthroplasty literature encompasses a myriad of indications for surgery, and specific pathology was not controlled for in the present study, nor in many cases of existing literature. As a result, comparisons are often made across heterogenous disease states, which can produce mixed complication rates and ROM outcomes, limiting the ability to draw accurate conclusions and comparisons. Postoperative rehabilitation protocol differences could also not be controlled for, as there is literature precedent of patients being allowed to initiate ROM exercises after TS-approach elbow arthroplasty sometimes three months earlier than after TD approaches: a factor that may have influenced the present study's observed ROM outcomes.^3^ Furthermore, many existing studies fail to distinguish TS and TD approaches when reporting outcomes, adding more challenges to literature comparison. In cases where separation was present and notable differences were observed between the existing literature and the findings of the present study, such insights may still fall within the ranges of standard statistical variation, denoting potentially smaller clinical differences than perceived. Given the narrative nature of outcome comparisons and the limited available data, especially in instances of relatively short follow-up, such claims must be interpreted with added caution. Accordingly, future large-scale studies of greater methodological rigor, quality, and focus on technique comparisons in elbow arthroplasty are warranted. Finally, heterogeneity in data reporting across included studies, such as differences in outcome definition between minor and major complications, or instances of incomplete data presentation, posed additional limitations to more advanced statistical syntheses, literature comparisons, and strengths of conclusions made. Despite these limitations, however, this study is the first systematic review to examine TS elbow arthroplasty outcomes, and its contemporary study selection criteria offer an updated, translational dataset reflective of the largest cohort to date, supplementing the necessary information for patient management and surgical decision making.

## Conclusion

This systematic review investigated current outcomes in TS elbow arthroplasty published since 2020, identifying 5 TEA studies comprising 195 patients and 3 EHA studies comprising 52 patients. Demographically, TS elbow arthroplasty groups were found to be very similar to that of TD elbow arthroplasty literature, as were their flexion and prosupination arc ROM results. As anticipated, TS-approach extension deficit outcomes were modestly superior when narratively compared to TD approaches. Complications were a distinguishing factor: TS-TEAs demonstrated acceptable overall complication, infection, and triceps insufficiency rates, whereas TS-EHAs exhibited concerns regarding overall complication and heterotopic ossification rates. Further research comprised of larger sample sizes is needed to better control for arthroplasty-indicating pathology, improve cross-technique comparison homogeneity, and standardize outcome reporting for profiling ROM and complication rates. Therefore, patients and surgeons should carefully weigh the risks and benefits of TS elbow arthroplasty when determining the most appropriate and practical surgical approach for clinical care.

## Disclaimers:

Funding: No funding was disclosed by the authors.

Conflicts of interest: The authors, their immediate families, and any research foundations with which they are affiliated have not received any financial payments or other benefits from any commercial entity related to the subject of this article.
